# Benefit from preoperative radiotherapy in rectal cancer treatment: disease-free patients' and oncologists' preferences

**DOI:** 10.1038/sj.bjc.6603954

**Published:** 2007-09-11

**Authors:** A H Pieterse, A M Stiggelbout, M C M Baas-Thijssen, C J H van de Velde, C A M Marijnen

**Affiliations:** 1 Department of Medical Decision Making, University Medical Center Leiden, Leiden, the Netherlands; 2 Department of Surgery, University Medical Center Leiden, Leiden, the Netherlands; 3 Department of Radiotherapy, Netherlands Cancer Institute, Amsterdam, the Netherlands; 4 Department of Clinical Oncology, University Medical Center Leiden, Leiden, the Netherlands

**Keywords:** treatment preference, adaptive conjoint analysis, treatment tradeoff method, treatment outcomes

## Abstract

Preoperative radiotherapy (PRT) in resectable rectal cancer improves local control but increases probability of faecal incontinence and sexual dysfunction. Consensus was reached in 2001 in the Netherlands on a guideline advising PRT to new patients. Purpose was to assess at what benefit oncologists and rectal cancer patients prefer PRT followed by surgery to surgery alone, and how oncologists and patients value various treatment outcomes. Sixty-six disease-free patients and 60 oncologists (surgical, radiation, medical) were interviewed. Minimally desired benefit from PRT (local control) was assessed using the Treatment Tradeoff Method. Importance of survival, local control, faecal incontinence, and sexual dysfunction in determining treatment outcome preferences was assessed using Adaptive Conjoint Analysis. The range of required benefit from PRT varied widely within participant groups. Seventeen percent of patients would choose PRT at a 0% benefit; 11% would not choose PRT for the maximum benefit of 11%. Mean minimally desired benefit excluding these two groups was 4%. For oncologists, the required benefit was 5%. Also, how strongly participants valued treatment outcomes varied widely within groups. Of the four outcomes, participants considered incontinence most often as most important. Relative treatment outcome importance differed between specialties. Patients considered sexual functioning more important than oncologists. Large differences in treatment preferences exist between individual patients and oncologists. Oncologists should adequately inform their patients about the risks and benefits of PRT, and elicit patient preferences regarding treatment outcomes.

Rectal cancer affects approximately 2500 new patients yearly in the Netherlands (Comprehensive Cancer Centers, the Netherlands, http://www.ikcnet.nl/IKZ/index.php?id=1646&nav_id=160&regio_id=124, accessed on 14 December 2006), and 5-year survival is around 65% (Peeters *et al*, 2006). A Dutch multimember trial (total mesorectal excision (TME) trial) showed neoadjuvant radiotherapy (5 × 5 Gy) followed by TME surgery to improve local control compared to TME surgery alone in resectable rectal cancer patients at 2-year follow-up, with no survival benefit (Kapiteijn *et al*, 2001). A Swedish population-based study showed similar results (Dahlberg *et al*, 1998). As local recurrences result in painful and severe disabling symptoms that are difficult to treat, a national guideline was agreed upon in 2001 based on these results, advising preoperative radiotherapy (PRT) for all resectable rectal cancer patients. Five-year follow-up trial data confirmed a reduced recurrence rate (from 11 to 6%), still with no survival benefit (Peeters *et al*, 2006). Irradiated patients reported higher rates of faecal incontinence compared to non-irradiated patients at 2- and 5-year follow-up (Marijnen *et al*, 2005; Peeters *et al*, 2005). The Swedish Rectal Cancer Trial showed comparable results at a mean of 6 years follow-up (Dahlberg *et al*, 1998). Moreover, irradiated patients showed higher rates of sexual dysfunction at 2-year follow-up (Marijnen *et al*, 2005).

Treating all eligible patients with PRT implies that many will be treated unnecessarily. It is presently questionable how strongly oncologists believe the benefit from PRT on local control to outweigh the risks. At the time of our study, PRT was the standard treatment in the Netherlands, but it was expected that the guideline might undergo changes, due to the publication of the above studies of Marijnen *et al* (2005) and Peeters *et al* (2005). Additionally, patients' views on the underlying risk/benefit tradeoff have never been assessed. Our study was undertaken to assess (a) at what benefit patients and oncologists prefer PRT followed by surgery to surgery alone, (b) how patients and oncologists value various treatment outcomes, and (c) whether characteristics of patients and oncologists affect preferences. The method used to value treatment outcomes is novel. This study may therefore serve as a model for similar tradeoffs in other cancer treatment decisions, as tradeoffs between tumour control and quality of life are abundant in oncology.

## MATERIALS AND METHODS

### Study population

To obtain as much variance in preferences as possible, we stratified sampling by PRT, type of surgery (permanent stoma or not), and incontinence/sexual dysfunction (if the tumour is close to the anal verge, rectal cancer surgery results in a permanent stoma; patients without a stoma face the risk of faecal incontinence). We aimed to include 60 disease-free rectal cancer patients, half of whom had been treated with surgery alone and half with PRT followed by surgery. In both groups, half should have reported sexual dysfunction at 2-year follow-up, and two-third should be without a permanent stoma. Of them, half should have reported faecal incontinence at 5-year follow-up. We randomly selected these patients from those participants of the TME trial who had agreed at 5-year follow-up to being approached for further research, and who were below age 90. Ninety-four eligible patients were approached. Four patients could not be reached. Nine had (had) other types of cancer or recurrent disease and were excluded. Of the remaining 81 patients, 70 (86%) agreed to participate. Reasons for refusal were the psychological burden (*N*=8), physical burden (*N*=1), time investment (*N*=1), or unknown (*N*=1).

We further aimed to include 60 oncologists. Seventy eligible oncologists were approached and 61 (87%) agreed to participate (25 surgical, 26 radiation, 10 medical). Reasons for refusal were time constraints (*N*=3), considering participation not meaningful (*N*=1 medical oncologist), being retired (*N*=1), unknown (*N*=1), or because oncologists could not be reached despite repeated attempts (*N*=3). Participating oncologists were specialised in gastroenterology and worked in academic and non-academic institutions. Participants were informed about the study by letter, and then asked for participation by telephone.

### Procedure

Individual face-to-face interviews were held at their home (patients) or institution (oncologists). At the beginning of the interview, patients were asked for informed consent. The interviewers were trained and adhered to a strict interview script. Furthermore, the Adaptive Conjoint Analysis (ACA) was fully computerised. For these reasons, possibility of an interviewer effect was minimal. The Medical Ethical Board of the Leiden University Medical Center approved the study. Socio-demographic, medical history (patients), and work-related (oncologists) details were assessed by self-report questionnaire, a week before the interview.

### Measures

**Figure 1:**
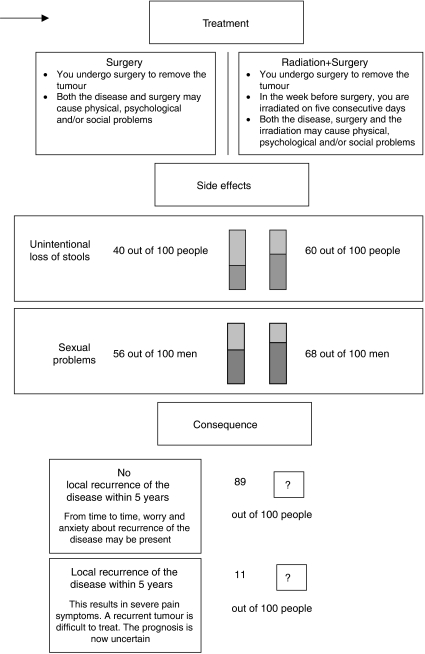
Consecutive information (treatment options, side effects, and consequence) presented with the TTM (male patient).

Minimally desired absolute benefit (local control) from PRT was assessed using the Treatment Tradeoff Method (TTM) (Llewellyn-Thomas, 1997). The TTM presented sequentially a short description of the two treatments and the respective probabilities of side effects as had been established at 2-year (sexual dysfunction; Marijnen *et al*, 2005) and 5-year (incontinence; Peeters *et al*, 2005) follow-up. Figure 1 depicts the male version. In the female version, numbers for sexually active patients were 15 (surgery) and 22 (PRT+surgery) out of 100 for dysfunction (Sexual dysfunction includes sexual inactivity. The overall numbers for dysfunction in female patients were 52% in the non-irradiated group and 57% in the irradiated group. We chose to present numbers for sexually active patients only, as too many respondents may otherwise disregard the difference between the treatment groups on this aspect). The interviewer explicitly stated that 5-year survival was the same following either treatment. Next, the probability of 5-year local control after surgery alone was presented (Peeters *et al*, 2005, 2007). The probability of 5-year local control with PRT was then varied and participants were asked each time which treatment they preferred: first (patients only) local control in 89 out of 100 patients (i.e. no benefit), then local control in 100 out of 100 patients (i.e. maximum benefit), and local control in 95 out of 100 patients. Participants' minimally desired benefit was searched by systematically bracketing the numbers further within the range of 89 out of 100 to 100 out of 100.

Next, relative importance of treatment outcomes was assessed using a computer-administered ACA task. This method has not been used in oncology as of yet, but has been applied in studies involving rheumatology (Fraenkel *et al*, 2001, 2004a, 2004b, 2006), HIV (Beusterien *et al*, 2005), and major abdominal surgery patients (Gan *et al*, 2004). Elsewhere we have described methodological aspects of this new method in an oncology setting (Pieterse *et al*, 2007). Preferences for various probabilities of 5-year survival, 5-year local control, faecal incontinence, and sexual dysfunction were elicited by asking participants to tradeoff combinations of these. Survival was included to make results more generalisable, as adjuvant treatment may improve survival after longer follow-up and in other cases. It was explicitly stated that survival and local control should be viewed as independent outcomes. Ranges of probability estimates included those that were established at 2-year (sexual dysfunction; Marijnen *et al*, 2005) and 5-year (survival, local control, incontinence; Peeters *et al*, 2005, 2007) follow-up (Table 1). Treatment modality was not specified. Separate versions of the ACA questionnaire were built for male and female patients.

**Figure 2:**
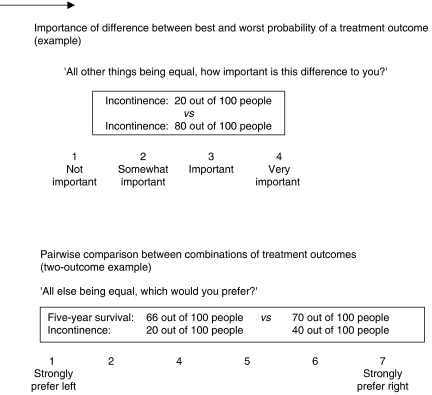
Adaptive conjoint analysis questionnaire.

The ACA questionnaire first asked participants to rate how important they considered the difference between the best and worst probability of each outcome (all else being equal) on a four-point scale (Fraenkel *et al*, 2001, 2004a, 2004b) (Figure 2). Next, participants were asked to rate 14 pairs of combinations of outcomes, where each combination consisted of two or three outcomes. Participants were asked to indicate their preference and strength of preference on a seven-point scale (Beusterien *et al*, 2005) (Figure 2). ACA customises the task to individual participants by presenting combinations representing tradeoffs that participants consider as increasingly relevant, based on their previous replies. The ACA analysing program (version 5.2.2, Sawtooth Software, Sequim, WA, USA) estimates how highly participants value each outcome probability and computes participants' relative importance score for each outcome, expressed as a percentage (importance scores add up to 100%) (ACA 5.0 Technical Paper, 2005). This score indicates the extent to which one outcome explains a participant's preference in choosing between outcome states, relative to the other outcomes. Due to the computation algorithm, the importance score for a specific outcome tends to be positively related to its range of probability. Importance scores should therefore not be viewed as absolute preferences. In an earlier analysis of the data, we showed that mean group importance could reliably be assessed over time (Pieterse *et al*, 2007).

In both TTM and ACA, quantitative frequency formats were used to facilitate understanding (Gigerenzer, 1996) and patients were asked to imagine that they had recently been diagnosed with cancer. Patients answered the gender-matching version of each task. Oncologists answered the TTM task for both male and female patients (in randomised order), and answered one randomly selected version of the ACA task.

### Statistical analyses

Descriptive statistics were used to describe participants and their minimally desired absolute benefit from PRT (TTM). Benefit was compared for socio-demographic, treatment- (patients), and work-related (oncologists) characteristics using the Kruskal–Wallis tests, independent *t*-tests, or Pearson's correlations. Treatment preference was said to be surgery alone if participants required a benefit exceeding the absolute 5% increase in probability of local control (Peeters *et al*, 2007). Patients' and oncologists' preferences were compared using a *χ*^2^-test. Regarding the ACA, we assessed whether participants' valuation of outcome probabilities was in agreement with probabilities from best to worst, within each of the four outcomes. Participants who valued the lowest probability of a good outcome highest were excluded from further analyses. Descriptive statistics were used to describe participants' relative importance, and these were compared using ANOVA and independent *t*-tests. *Post hoc* comparisons were analysed using the Bonferroni test. All significance testing was performed two-tailed at *α*=0.05.

## RESULTS

### Participants

Table 2 lists demographic, treatment- (patients), and work-related (oncologists) details. Of the 70 patients, 66 (94%; 45 male, 21 female) completed the TTM- and ACA tasks. One patient had so many difficulties in comprehending numbers that he did not complete the TTM task and was not asked to perform the ACA task. Others did not perform the ACA task due to a computer failure (*N*=1) or finding the task too difficult (*N*=2).

One radiation oncologist completed the questionnaire and withdrew from the interview because of time constraints. All surgeons as well as 14 out of 25 radiation and 9 out of 10 medical oncologists were male.

### Minimally desired benefit from PRT

**Figure 3:**
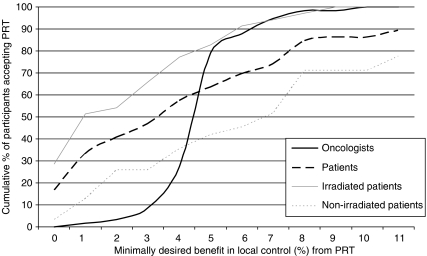
Cumulative proportion of oncologists (*N*=58) and patients (*N*=66) preferring PRT according to minimum percentage of benefit in local control. Numbers of patients do not add up to 100% because of those never preferring PRT.

Figure 3 shows the cumulative proportion of participants preferring PRT according to minimum benefit. Seven (11%) patients would not choose PRT for the maximum benefit of 11%. Eleven (17%) patients would choose PRT at no benefit. In the other 48 patients, average minimally desired benefit was 4.4% (95% CI 3.6–5.3; range 1–11). Overall, average minimally desired benefit was not significantly different for male and female patients (3.3±2.8 *vs* 4.4±3.8, *P*=0.20), and was significantly lower for irradiated patients (2.6±2.6 *vs* 5.1±3.3, *P*<0.001) and those with a stoma (2.1±2.4 *vs* 4.5±3.2, *P*=0.01).

One medical oncologist would not advise PRT to male patients, and only for a 7% benefit to female patients. One surgical oncologist would advise PRT to male patients for 6% benefit, but could not decide for female patients. In the remaining oncologists, required benefit from PRT for male *vs* female patients was identical for 54 out of 57 (95%, 1 missing value) and 1% higher or 1–2% lower for male patients in other cases. Results were therefore pooled. On average, minimally desired benefit was 5.0% (95% CI, 4.6–5.4; range 1–10). Most frequently cited (by *N*=30) desired benefit was 5% (Figure 3). Minimally desired benefit was, respectively, 4.7±1.2, 5.0±0.5, and 5.3±1.8 for radiation, medical, and surgical oncologists (*P*=0.56). Minimally desired benefit was not correlated with age, time since specialisation, current workplace, having ever supervised internships or residencies, or having ever been part of a guideline committee (data not shown).

For a 5% benefit in local control, there was a trend for oncologists to prefer PRT more often than patients (79 *vs* 64%, *P*=0.06). Oncologists preferred PRT significantly more often than non-irradiated patients (79 *vs* 42%, *P*<0.001) and about as often as irradiated patients (79 *vs* 83%, *P*=0.68).

### Relative importance of treatment outcomes

Five (4%) participants conferred the highest value to the worst outcome probability in one of the outcomes and were excluded from further analyses. Incontinence was the most important treatment outcome for 29 (47%) patients and 24 (41%) oncologists, followed by local control for 21 (34%) patients and 20 (34%) oncologists, survival for 7 (11%) patients and 12 (20%) oncologists, and sexual dysfunction for 5 (8%) patients and for 3 (5%) oncologists.

**Figure 4:**
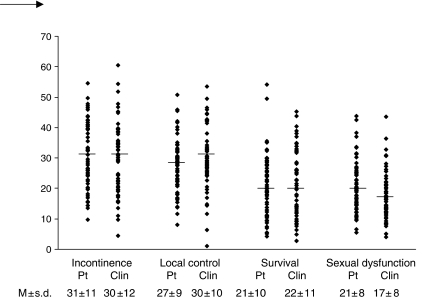
Individual importance scores (range 0–100) of treatment outcomes in patients and clinicians. M=mean; s.d.=standard deviation; Pt=patient; Clin=clinician. One oncologist and four patients were excluded from the analyses because they valued the worst probability of one of the treatment outcomes highest compared to the other outcome probabilities of that outcome.

Figure 4 shows mean, standard deviation, and range of relative treatment outcome importance. Relative importance did not differ between male and female patients, irradiated and non-irradiated patients, or between those with and without a stoma. An effect of the experience of the side effects of treatment on the relative importance of those side effects was not seen either. No differences were seen between patients who did and who did not suffer from incontinence with respect to the importance attached to incontinence, and no differences were seen for sexual dysfunction between patients who did and who did not indicate sexual problems.

Mean importance was significantly different between specialties for local control (*P*=0.01), survival (*P*=0.02), and sexual dysfunction (*P*=0.024). Radiotherapists considered local control more important than medical oncologists (35±9 *vs* 24±8, *P*=0.02) and surgeons (28±11, *P*=0.04). Surgeons considered sexual dysfunction more important than radiotherapists (20±9 *vs* 14±5, *P*=0.02). There was a trend (*P*=0.05) for medical oncologists to consider survival more important than surgeons (28±9 *vs* 17±12). Relative importance of treatment outcomes for oncologists did not differ according to patient gender or oncologists' background characteristics (data not shown), except that clinicians who had supervised tended to consider local control as more important than clinicians who had not (36±9 *vs* 29±10 *P*=0.05).

Mean relative importance of probability of sexual dysfunction was significantly higher for patients than for oncologists (21±8 *vs* 17±8, *P*=0.02).

## DISCUSSION

The Dutch TME trial showed that short-course PRT improves local control in resectable rectal cancer patients, with no survival gain (Peeters *et al*, 2007). Given the high rates of local control with surgery alone (Peeters *et al*, 2007), 90–95% of patients are unnecessarily treated with PRT. Radiotherapy has been shown to induce major side effects, including faecal incontinence (Marijnen *et al*, 2005; Peeters *et al*, 2005), sexual dysfunction (Marijnen *et al*, 2005), small bowel obstruction (Birgisson *et al*, 2005a), and development of secondary tumours (Birgisson *et al*, 2005b). The awareness of side effects often leads to discussions in multidisciplinary oncology meetings about the necessity of PRT for certain patients. One would expect that a small probability of benefit and large probabilities of side effects would call for the input of the patient in the process of decision-making. However, in the Netherlands, these probabilities are often not explicitly discussed with the patient, and the decision about PRT is even less frequently left to the patient. We therefore performed this study to evaluate patients' and oncologists' preferences for preoperative treatment. Investigating how new rectal cancer patients value the tradeoff between local control and side effects is difficult in the Netherlands, where neoadjuvant radiation is the standard treatment. We therefore recruited disease-free rectal cancer patients treated in the TME trial as an alternative, enabling us to assess views from patients with and without experience with PRT and side effects.

The TTM methodology showed that patients preferred to be irradiated when the mean gain in local control was about 5%. The range was considerable (0–11%) though, highlighting the need for a discussion of the pros and cons of PRT with every patient with resectable rectal cancer.

One of the drawbacks of this study is the use of already treated patients, who have been disease-free for over 5 years. To these patients, 5-year survival and 5-year local control rates will have another connotation than to recently diagnosed patients. Since oncologists in the Netherlands are reluctant to discuss these absolute rates explicitly with patients, we deemed it impossible to use the methods with recently diagnosed patients. We were aware that our retrospective interviews would incorporate the biases generally seen in the literature, but by asking all types of patients, both with and without treatment experience, and with and without symptoms, we wished to obtain an impression of all possible attitudes towards the tradeoffs involved. Patients have been shown to have a strong preference for the therapy they have undergone (Yellen *et al*, 1994; McQuellon *et al*, 1995; Stiggelbout *et al*, 1996; Lindley *et al*, 1998; Jansen *et al*, 2001), possibly embracing it psychologically as the best possible for them. Indeed, patients in this study who underwent PRT desired a lower benefit from irradiation than non-irradiated patients. A significant minority preferred PRT even if the treatment was non-beneficial and harmful, in accordance with studies on adjuvant chemotherapy (Ravdin *et al*, 1998; Jansen *et al*, 2001) and adjuvant radiotherapy (Palda *et al*, 1997). Again, this preference may result from cognitive justification, but may also be grounded in expecting or having experienced non-clinical benefits, including a sense of control over one's situation (Levine *et al*, 1988), persistent belief in treatment benefit (Palda *et al*, 1997), or avoiding negative feelings including regret over having refused treatment (Palda *et al*, 1997). One should take this into account when judging the absolute benefit that our patients required from treatment. The number for newly diagnosed patients will likely lie in between the means of our two patient groups.

On the basis of the frequent discussions in multidisciplinary team meetings, we hypothesised that required benefit from PRT would differ between specialties. However, our results showed that most oncologists, regardless of specialty, prefer PRT when the gain in local control is 5%. This is a generally accepted benefit in Dutch oncologists regarding adjuvant treatment in general (Bontenbal *et al*, 2000). The fact that the TME trial showed 5% benefit at 5-year follow-up (Peeters *et al*, 2007) probably explains why PRT is so rarely presented to the patient as a choice or a decision to be made. Nevertheless, the range in local control we established for patients to prefer PRT demonstrates that lack of choice may not be justified.

Regarding relative preferences for treatment outcomes, findings were highly comparable between patients and oncologists. First, in both groups about 40% of participants considered faecal incontinence as the major drawback of rectal cancer treatment. The relative importance of tumour control, survival, and sexual functioning was also similar in both groups. Second, there was large between-subject variation in the importance of local control, survival, incontinence, and sexual functioning in determining participants' preferences for outcome states. This indicates that both individual patients and individual oncologists greatly vary in their perception of how tumour control, survival, and quality of life should be weighed in deciding upon the most preferable treatment.

The relative importance of the various outcomes of treatment was similar for irradiated and non-irradiated patients. This finding from ACA is in contrast to preferences as assessed using the TTM. It may be explained by the different cognitive processes invoked by the two tasks. In contrast to the TTM, the ACA does not identify treatment modality. Cognitive justification of a previous treatment experience, seen in the TTM, will therefore not strongly influence preferences.

ACA explicitly asks to evaluate tradeoffs between benefits and side effects, a task that is highly relevant to treatment decision-making in oncology. This method, which is novel to the field of oncology, is therefore promising, and indeed the large majority of our oncologists felt this to be the case (55% definitely, 27% possibly). Analysis of data we are currently gathering among patients (*N*=28) who have been treated more recently (*M*=1.8±1.1 years ago; range 0.4–5.6) suggests that for them incontinence, tumour control, survival, and sexual dysfunction determined in that order preferences for treatment outcome, as was the case for the patients in this study. Also, how important these outcomes were rated on average did not significantly differ from this patient group. These findings suggest that for disease-free patients who are still within 5 years of surgery, local control is not more important and quality of life not less important than to patients who have survived disease-free beyond 5 year.

Differences in the importance of the various treatment outcomes were seen between specialties. Tumour control was more important to radiation than to other oncologists, possibly because that is the benefit that can be gained from the treatment they offer. This finding is in accord with results showing that specialists tend to believe in the therapy they deliver, as was found for urologists and radiotherapists in the treatment of localised prostate cancer (Fowler *et al*, 2000), and for medical oncologists in adjuvant chemotherapy treatment for breast cancer (Stiggelbout *et al*, 2000). Sexual dysfunction was somewhat more important to surgical than to radiation oncologists. Additional analyses suggest that relative importance of sexual dysfunction cannot be explained by the difference in gender distribution between specialties, since the relative importance did not differ overall between male and female oncologists (data not shown). The small number of female oncologists, however, prevents us from drawing strong conclusions.

Sexual functioning was equally important to male and female patients, and more strongly determined patients' than oncologists' preferences. This result suggests that the harmful effect of treatment on this aspect of patients' lives deserves attention, and possibly to a larger extent than oncologists may think.

In conclusion, treatment preferences differ between individual patients, individual oncologists, and oncologists from different specialties treating the same patient. This inter-individual variability, coupled with the prospect of a long life expectancy after surgery, indicates that oncologists should provide patients with comprehensive information about benefits and side effects of neoadjuvant radiation, and elicit patients' outcome preferences in the process of treatment decision-making.

## Figures and Tables

**Table 1:** ACA treatment outcomes and outcome-probabilities (Frequencies out of 100 patients)

**Outcome**	**Explanation**	**Outcome probabilities (from best to worst)**			
Probability of 5-year survival (all patients)	This is the probability that the patient is still alive 5 years after the disease was detected. A 5-year survival of 50% means that after 5 years, 50 out of 100 patients are still alive. The other 50 people may have died due to the recurrence of the disease, but may also well have died from other causes such as a heart attack	70	66	65	—
Probability of five-year local control^a^ (all patients)	This is the probability that the tumour does not recur at the site that was operated on. If the tumour does recur at that site, it causes a lot of pain. It may in some instances be possible to treat it, but in others not. Often the prognosis is uncertain	99	94	89	—
Probability of faecal incontinence (all patients)	Incontinence in this interview refers to incontinence for stools and means unintentionally losing stools	20	40	60	80
Probability of sexual dysfunction (male patients)	You may think of problems with getting an erection (=erectile dysfunction) and with ejaculation, or of not being sexually active at all anymore	30	40	50	60
Probability of sexual dysfunction (female patients)	Dissatisfaction with sexuality usually results from not being able to enjoy sexual intercourse anymore because of pain or vaginal dryness	10	30	50	70

Abbreviation: ACA, Adaptive Conjoint Analysis.

a The expression ‘probability of local control’ was not used in patients but was explained as ‘probability that the tumour does not recur’.

**Table 2:** Participants' background details

**Participants**	***N* (%)**
*Patients (N*=*66)*	
Mean age, years±s.d. (range)	64±9.2 (41–84)
Mean time since surgery, years±s.d. (range)	8±1.0 (6–10)
Treatment	
Surgery	31 (47)
PRT+surgery	35 (53)
Permanent stoma	
Yes	25 (38)
No	41 (62)
Incontinence (non-stoma patients)	
Never	23 (56)
Sometimes	17 (41)
Often	1 (2)
Always	0
	
*Oncologists (N*=*60)*	
Mean age, years±s.d. (range)	48±7.3 (35–62)
Mean time since specialisation, years±s.d. (range)	13±8.1 (1–31)
Current institution	
Academic	14 (23)
Non-academic	46 (77)
Supervisor (ever)	
Yes	9 (15)
No	50 (85)
Member of a guideline committee (ever)	
Yes	13 (22)
No	47 (78)
Adherence to 2001 guideline^a^	
Overall yes	53 (90)
Yes, except for high tumours	2 (3)
No, not in general	4 (7)

Numbers do not add up to 60 in oncologists due to missing data.

a Reported rectal cancer treatment management within the oncologist's institution.
